# 2334. The effectiveness of bivalent COVID-19 booster among patients with hematologic malignancies

**DOI:** 10.1093/ofid/ofad500.1956

**Published:** 2023-11-27

**Authors:** Muneerah M Aleissa, Sonya Davey, Rebecca Rooks, Natalie E Izaguirre, Bridget Yates, Urwah Kanwal, Lindsey R Baden, Nicolas C Issa, Amy C Sherman

**Affiliations:** Brigham and Women's Hospital, Boston, Massachusetts; Brigham and Women's Hospital, Boston, Massachusetts; Brigham and Women's Hospital, Boston, Massachusetts; Brigham and Women's Hospital, Boston, Massachusetts; Brigham and Women's Hospital, Boston, Massachusetts; Brigham and Women's Hospital, Boston, Massachusetts; Brigham and Women's Hospital, Boston, Massachusetts; Brigham & Women's Hospital, Boston, Massachusetts; Brigham and Women's Hospital, Boston, Massachusetts

## Abstract

**Background:**

Patients with hematologic malignancies remain at higher risk for developing severe SARS-CoV-2 infections and have reduced immune responses to vaccines.

**Methods:**

A prospective observational study was conducted at Brigham and Women’s Hospital and Dana-Farber Cancer Institute from January 2021 to May 2023. Adult participants were included if they had a history of lymphoid malignancy (LM) or had received hematopoietic stem cell transplantation (HSCT). SARS-CoV-2 antibodies were measured before vaccination and every 2-3 months thereafter. COVID-19 vaccination history and SARS-CoV-2 infections (by report or positive PCR) were recorded longitudinally. Our primary outcome for this study was to evaluate the incidence of breakthrough SARS-CoV-2 infections after receiving a bivalent COVID-19 vaccine booster (Omicron/wildtype).

**Results:**

A total of 144 participants were included in the analysis. Of those, 98 (68%) received a bivalent COVID-19 booster. The median age was 68 (IQR 61 – 73) and 51 (52%) were male. The majority of participants 61 (62%) had LM and 37 (38%) underwent HSCT with a median time from transplant to first vaccine of 317 days (IQR 171 – 742). Overall, the incidence of breakthrough SARS-CoV-2 infections was 13 (13%) (Figure) with a median time from vaccination to breakthrough infection of 72 days (IQR 54 – 112). There were no SARS-CoV-2 related mortality or severe disease leading to hospitalization in our cohort.

Figure

**Figure:**
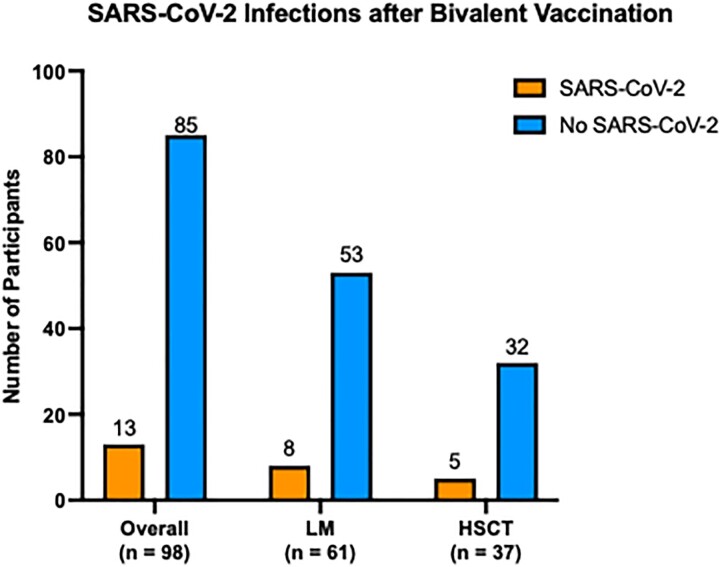

**Conclusion:**

Our analysis demonstrates a low incidence of breakthrough SARS-CoV-2 infections and no severe disease among those who received a bivalent mRNA vaccine, highlighting the potential protection of the bivalent booster against SARS-CoV-2 in patients with hematologic malignancies who had previously been vaccinated or boosted. Further studies are warranted to determine the optimal number and type of booster vaccinations needed to protect this vulnerable population.

**Disclosures:**

**Nicolas C. Issa, MD**, AiCuris: Grant/Research Support|Astellas: Grant/Research Support|Boehringer Ingelheim: Advisor/Consultant|Fujifilm: Grant/Research Support|GSK: Grant/Research Support|Merck: Grant/Research Support|Moderna: Grant/Research Support

